# Therapeutic Potential of Nutraceuticals and Dietary Supplements in the Prevention of Viral Diseases: A Review

**DOI:** 10.3389/fnut.2021.679312

**Published:** 2021-09-17

**Authors:** Saumya Singh, Prithwish Kola, Dalveer Kaur, Gisha Singla, Vibhu Mishra, Parmjit S. Panesar, Kumar Mallikarjunan, Meena Krishania

**Affiliations:** ^1^Center of Innovative and Applied Bioprocessing (CIAB), Sector-81 (Knowledge City), Mohali, India; ^2^Food Biotechnology Research Laboratory, Department of Food Engineering & Technology, Sant Longowal Institute of Engineering & Technology Longowal, Longowal, India; ^3^Food Science and Nutrition Department, University of Minnesota, Minneapolis, MN, United States

**Keywords:** nutraceuticals, viral diseases, functional foods, immunity, coronavirus, dietary supplements

## Abstract

Nowadays, despite enormous scientific advances, viral diseases remain the leading cause of morbidity worldwide, and their potential to spread is escalating, eventually turning into pandemics. Nutrition can play a major role in supporting the immune system of the body and for the optimal functioning of the cells of the immune system. A healthy diet encompassing vitamins, multi-nutrient supplements, functional foods, nutraceuticals, and probiotics can play a pivotal role in combating several viral invasions in addition to strengthening the immune system. This review provides comprehensive information on diet-based scientific recommendations, evidence, and worldwide case studies in light of the current pandemic and also with a particular focus on virus-induced respiratory tract infections. After reviewing the immune potential of nutraceuticals based on the lab studies and on human studies, it was concluded that bioactive compounds such as nutraceuticals, vitamins, and functional foods (honey, berries, etc.) with proven antiviral efficacy, in addition to pharmaceutical medication or alone as dietary supplements, can prove instrumental in treating a range of virus-induced infections in addition to strengthening the immune system. Milk proteins and peptides can also act as adjuvants for the design of more potent novel antiviral drugs.

## Introduction

A tremendous rise in virus-induced infections and the associated mortality rate has created the demand to come up with effective and safe antiviral drugs. Drug development in case of viruses is challenging due to the emergence of drug-resistant pathways, a limited number of targets, the rapid evolution of viral genes, and the appearance of new viral strains *via* mutations. WHO has reported around 22 different viral outbreaks in 2019 alone. From the newly emerged viral diseases like ebola virus (in the Republic of Uganda and the Democratic Republic of Congo), hantavirus (in the Republic of Panama and Argentine Republic), zika virus (in France), Middle East respiratory syndrome coronavirus (MERS-CoV) (in the Kingdom of Saudi Arabia, Oman, the United Arab Emirates, and Qatar) to well-known viral diseases like measles (in Madagascar, European Region, Western Pacific Region, Tunisia, Lebanon, and Pacific Island Countries and Areas), and dengue fever (in Jamaica, Reunion, France, Pakistan, the Republic of Sudan, Spain, and Afghanistan), the scenario of a viral outbreak is getting worse day by day. According to WHO, the biorisk reduction is mainly based on the use of the current scientific understanding related to the viral hemorrhagic fevers, zoonotic diseases, and epidemic-prone orthopoxviruses, to develop a direction for the control, treatment, and mitigation of the risk of these viral outbreaks regardless of the source. Symptomatic treatments and immunity boost are the most efficient methods as no specific drug is available for each of the viral diseases. Even though various new antiviral agents have been developed recently, still there are numerous constraints associated with the current antiviral treatments such as efficacy, safety, and high costs (1). Thus, in this scenario, the nonconventional therapy with vitamins, multi-nutrients, functional foods, nutraceuticals, and prebiotics can play a significant role to combat this rising threat. These agents are not only virucidal in action (inhibit viral replication and protein synthesis) but can also boost the natural immunity and increase the physiological condition of the human body (like replenishing glutathione (GSH) amount and controlling the amount of free radicals in the cells). Thus, it becomes difficult for the viruses to replicate inside the host body and the severity of the symptoms also decreases, which can lead to a low mortality rate and speedy recovery (2). The natural agents (like probiotics) can directly attach to the viruses in the gut, thus preventing the latter from adhering to the host cell by various mechanisms (steric hindrance, a receptor-specific competitive/non-competitive way, the attachment of other chemical agents to prevent binding, etc.) apart from eliciting an active immune response. The antiviral activity of various natural agents against coronavirus may also be done by the modulation of the immune responses (macrophages, dendritic cells, etc.), generation of cytotoxic (antiviral) chemicals and cells like nitric oxide- (NO-) free radicals, cytotoxic T lymphocytes from CD8+ T lymphocytes, T helper cells from CD4+ T lymphocytes, activation of phagocytosis, proliferation of B lymphocytes, etc. (3).

With an increase in age, human body starts to produce a smaller number of T cells due to thymus atrophy, thus making an individual susceptible to lethal infections. Therefore, nutrition can play a significant role in assisting the immune system and in optimizing cell functions, including the cells acting in the immune function of the body. Nutraceuticals serve to functionalize food and boost the idea of diet as daily nourishment in health-related aspects. In the present review, the efficacy of nutrients, nutraceuticals, probiotics, milk proteins, and functional foods for modulating immune responses and preventing viral infections/or ameliorating disease severity has been concisely summarized. In the following sections, the use of bioactive compounds in viral mechanisms, particularly SARS and respiratory viruses-related infections, has been discussed, which might help in facilitating preintervention either directly as supplements/enriched foods or in combination with pharmaceutical medication (4).

## Nutraceutical Supplements for Viral Infections

**Figure 1:**
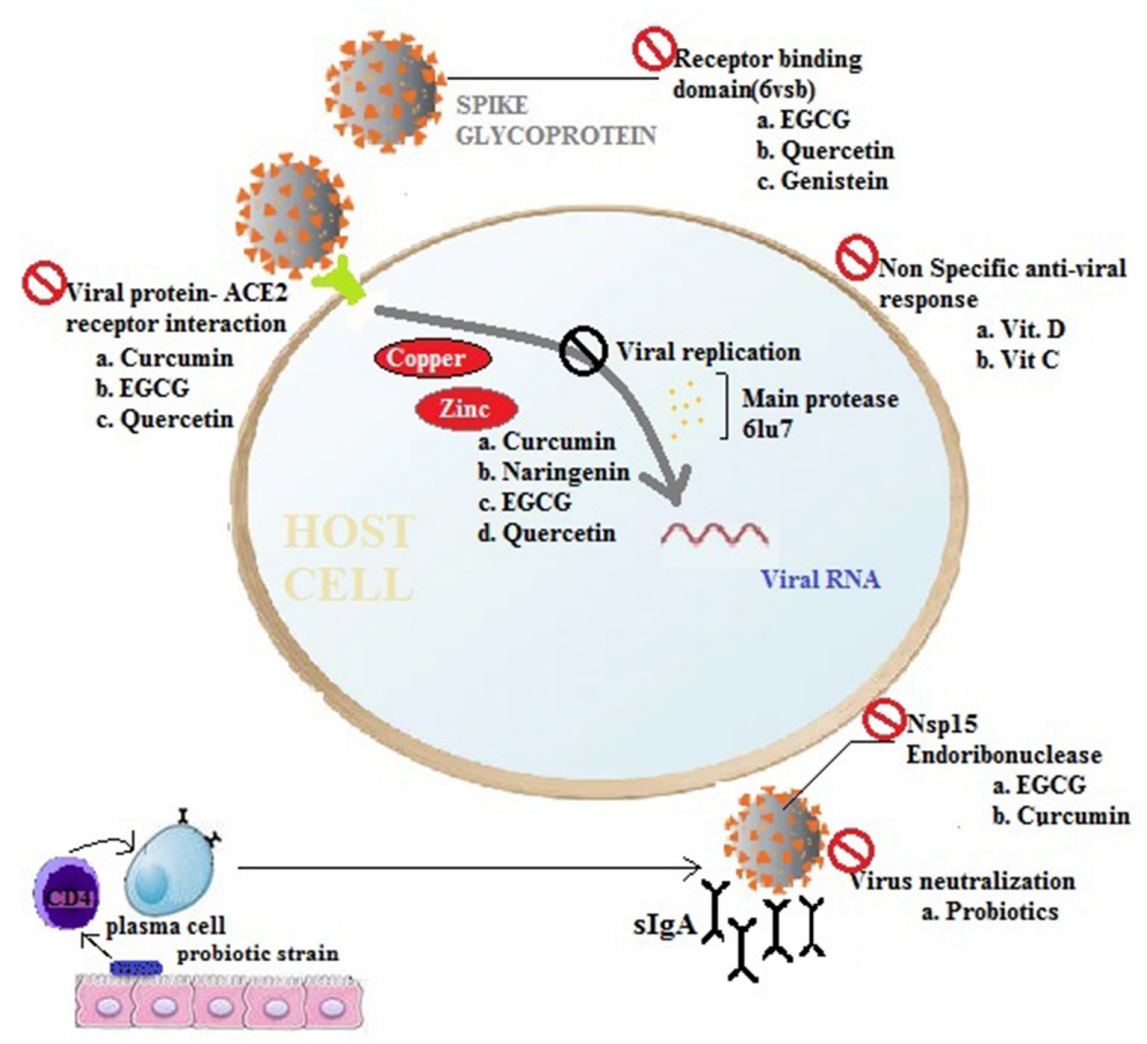
The potency of nutraceuticals and functional foods for preventing viral infections. Pre-intervention as supplements or in combinations with nutraceuticals and nutrient supplements modulate immune responses and prevent viral infections or ameliorate disease severity by acting at different stages of a virus invasion pathway (Nsp15, SARS-CoV-2 endoribonuclease; 6vsb, 2019-nCoV spike glycoprotein; 6lu7, SARS-CoV-2 main protease, ACE2, receptor for SARS-CoV-2; EGCG, Epigallocatechin gallate).

Nutraceuticals have antiviral, anti-inflammatory, and immunomodulatory effects, such as resveratrol (5), quercetin (6), curcumin (7), epigallocatechin gallate (EGCG) (8), N-acetyl cysteine (NAC) (9), and palmitoylethanolamide (PEA) (10) (as shown in Table 1). The antiviral activities of these nutraceuticals against the group of coronaviruses (like SARS-CoV-2 and COVID-19) are mainly based on their anti-inflammatory (the inhibition of NLRP3 inflammasome-mediated IL-beta production and inhibition of the pro-inflammatory cytokines) effect along with their viral replication inhibition property by regulating COVID-19 main protease (M^pro^) (15). The viral pathogenesis is controlled, and a symptomatic relief has been provided by these agents. Figure 1 shows a general pictorial representation of the modes of action of the selected nutraceuticals against viruses. Various nutraceuticals having an antiviral potential along with their modes of action have been discussed in the following sections.

**Table 1:** Compilation of antiviral properties and effects of selected nutraceuticals.

**Name**	**Virus**	**Mechanism of action**	**Effect**
Resveratrol (5, 11)	Influenza virus	1. The active blocking of nucleocytoplasmic translocation of viral ribonucleoproteins in MDCK cells 2. Inhibition of protein kinase C associated mechanism.	Inhibition of *in-vitro* and *in-vivo* viral Replication and protein expression
	Epstein-Barr Virus	1. Inhibition of EBV early antigen induction (through Raji cells), EBV lytic cycle, transcription genes and proteins, Rta, Zta, and diffused early antigen (EAD), EBV immediate-early protein: BRLF1 and BZLF1 promoters, transcription factors NF-*κβ* and AP1. 2. Downregulation of antiapoptotic proteins: Mc 1 STAT-3, miR-155, and miR-34a 3. Reduction in ROS production	1. Decrease in papilloma production, virion production 2. Inhibition of viral protein synthesis and transformation in human B-cells
	Herpes Simplex Virus	1. Decreased production of early viral protein ICP-4. 2. Induce the rapid and sustained release of ROS. 3. Inhibition of NF-*κβ*, extracellular signal-regulated kinases/mitogen-activated protein kinases (Erk/MAPK), immediate-early, early, and late HSV genes.	1. Reversible, dose-dependent inhibition of virus replication *In-vitro* and *in-vivo* 2. Prevention of viral reactivation in neuron cells, cutaneous lesions in abraded skin and vaginal lesions,
	Respiratory Syncytial Virus	1. Modulation of toll-like receptor 3 expression 2. Inhibition of toll/IL-1R domain-containing adaptor inducing IFN (TRIF) signaling, matrix metalloproteinase 12 (MMP-12), TANK binding kinase 1 (TBK1) protein expression, TNF-α, IL-2, IL-6, and nerve growth factor (NGF) secretion 3. Induction of muscarinic 2 receptor (M2R) and upregulation of sterile-α- and armadillo motif-containing protein (SARM) expression	1. Reduction in the level of interferon-gamma (IFN-γ) 2. Decreased number of inflammatory cells, reduction of inflammation reflex and airway inflammation. 3. Inhibition of viral replication
	Human Immunodeficiency Virus (HIV)	1. Inhibition DNA synthesis during the reverse transcription process 2. Activation of lytic cycle of HIV-1 *in vitro*;	1. Inhibition of HIV-1 replication *in-vitro* 2. attenuation of the Tat-induced HIV-1 LTR trans activation *in-vitro*
	Varicella Zoster Virus (VZV)	1. Reversible, dose-dependent inhibition of MRC-5 cells 2. Decrease the synthesis of intermediate early protein (IE 62)	Inhibition of VZV replication *in vitro*
	Enterovirus 71	1. Phosphorylation of proinflammatory cytokines (IKKα, IKKβ, IKKγ, IKBα, NF-*κβ* p50, and NF-*κβ* p65) 2. Inhibition of IL-6 and TNF-α Secretion	Inhibition of viral protein (VP-1) synthesis
	Duck Enteritis Virus	1. Inhibition of pro-inflammatory mediators (IL-1α, IL-6, and TNF-α), chemokines (CXCL10 and CCL4) secretion 2. Suppression of NF-*κβ* and interferon regulating factor (IRF-3)	1. Inhibition of viral replication, protein synthesis *in-vitro*. 2. Reduction of cellular oxidative damage.
	Human Metapneumonia Virus	1. Suppression of proinflammatory mediators (IL-1α, IL-6, and TNF-α) and chemokines (CXCL10 and CCL4) secretion 2. Inhibitory effect on NF-*κβ* and interferon regulating factor (IRF-3)	1. Inhibition of viral replication 2. Reduction of cellular oxidative damage and oxidation stress
	African Swine Fever Virus	Inhibition of protein synthesis and virion formation	Inhibition of viral replication *in-vitro*.
	Human Rhinovirus	1. Suppression of HRV-induced expression of ICAM-1 2. Inhibition of IL-6, IL-8, and RANTES secretion.	Anti-inflammatory effect
	Cytomegalovirus	Inhibition of activated epidermal growth factors (EGF), phosphatidylinositol-3-kinase signal transduction, NF-*κβ* and Sp1 transcription factors	Inhibition of HCMV replication and viral protein synthesis *in vitro*
	Polyomavirus	Blocking of DNA synthesis in a dose dependent manner	Inhibition of viral replication *in-vitro*
Quercetin (6, 12)	Duck Enteritis Virus	Along with Resveratrol, it suppressed proinflammatory mediators (IL-1α, IL-6, and TNF-α) and chemokines (CXCL10 and CCL4) secretion	Lowering of cellular oxidative damage
	Human Metapneumonia Virus	Along with Resveratrol it inhibits secretion of pro-inflammatory mediators (IL-1α, IL-6, and TNF-α) and chemokines (CXCL10 and CCL4)	Reduction of cellular oxidative damage
	Herpes simplex virus type 1 (HSV-1)	Along with TNF, quercetin increases the activity of IFN-β and up-regulates the IFN-β Production	Potentiates the dose dependent inhibitory effect of TNF on viral replication.
	Vesicular stomatitis virus (VSV)	Along with TNF, quercetin increases IFN-β activity and up-regulates the production of IFN-β.	Potentiates the dose dependent inhibitory effect of TNF on viral replication
	Encephalomyocarditis virus (EMCV)	Along with TNF, quercetin increases the action of IFN-β and up-regulates the production of IFN-β.	Potentiates the dose dependent inhibitory effect of TNF on viral multiplication
	Parainfluenza virus type 3 (Pf3)	Inhibits the DNA replication *in-vitro*	dose-dependent reduction in the infectivity of virus
Curcumin (7, 8)	Herpes simplex virus type 1 (HSV-1)	Down regulation of the immediate early (IE) genes.	Inhibition of HSV-1 replication
	Human Immunodeficiency Virus (HIV)	Obstruction of HIV-1 LTR-directed gene expression, Tat-assisted transactivation (Tat protein acetylation) of HIV-1 LTR, HIV-1 and HIV-2 proteases, HIV-1 Integrase	Inhibition of proviral DNA formation, functional protein formation from viral polyprotein and integration of proviral DNA into host DNA
	Influenza Virus	Inhibition of NF-κβ signaling	Inhibition of hemagglutination and viral propagation
	Hepatitis B virus	Increase in the level of p53	Inhibition of viral DNA replication
	Hepatitis C virus	Inhibition of the Akt-SREBP-1 pathway	Inhibition of viral DNA replication
	Coxsackievirus	Dysregulation of ubiquitin-proteasome system (UPS)	1. Inhibition of viral DNA replication, RNA expression 2. Protection against virus-induced apoptosis and cytopathic activity
	Japanese encephalitis virus (JEV)	1. Modulation of cellular levels of stress-related proteins and restoration of membrane integrity 2. Reduction of pro-apoptotic signaling molecules and ROS at cellular level	Provides neuroprotective effect
	Adult T-cell leukemia (ATL)	1. Suppression of DNA binding, transcriptional effect of AP-1 in HTLV-1-infected T-cell lines and JunD protein expression	1. Induction of cell cycle arrest and apoptosis 2. Inhibition of HTLV-1 replication in infected T-cell
EGCG (8)	Influenza Virus	The active blocking of nucleo-cytoplasmic translocation of viral ribonucleoproteins in MDCK cells	Dose dependent inhibition of virus
	Human Immunodeficiency Virus (HIV)	Inhibition of α-glucosidase.	Decrease the infectivity of virus
	Hepatitis C virus (HCV)	Inhibition of NS3/4A protease	1. Inhibition of virus maturation 2. Decrease in pathogenicity
	Herpes simplex virus (HSV)	Along with TNF, quercetin increases the action of IFN-β and upregulates the production of IFN-β	Potentiates the dose dependent inhibitory effect of TNF on viral replication.
	Enterovirus (EV)	1.Inhibition of Viral DNA replication in G6PD-deficient cells. 2. Reduction of EV associated cellular oxidative stress	1. Inhibition of infectious progeny virion formation. 2. Decrease of viral propagation
NAC (9, 13)	Pneumococcal meningitis	1. Scavenging Reactive Oxidation Species 2. Inhibition of inflammatory cytokines	1. Prevention of intracellular oxidation stress. 2. Prevention of Viral Pathogenesis
	Hepatitis C virus (HCV)	1. Scavenging Reactive Oxidation Species 2. Inhibition of inflammatory cytokines	1. Prevention of intracellular oxidation stress. 2. Decrease in viral pathogenesis
	Swine flu (H1N1) virus	1. Inhibit the down regulation of pulmonary catalase, glutathione and superoxide dismutase 2. Scavenging Reactive Oxidation Species	1. Prevention of intracellular oxidation stress. 2. Prevention of Viral Pathogenesis
	Bird Flu (H5N1) virus	Inhibition of the pro-inflammatory cytokines (e.g., TNF-α), chemokines (e.g., IP10) secretion from primary human macrophages *in –vitro*	1. Prevention of intracellular oxidation stress. 2. Prevention of Viral Pathogenesis
	Human Immunodeficiency Virus (HIV)	1. Scavenging Reactive Oxidation Species 2. Deactivation of cellular transcription factor (NFK-β) 3. Inhibition of the upregulation of pro-inflammatory cytokines (e.g., tumor necrosis factor-a) secretion and HIV-1 LTR-directed gene expression	1. Prevention of intracellular oxidation stress. 2. Prevention of Viral Pathogenesis 3. Inhibition of HIV-transcription and replication
PEA (10, 14)	Influenza and common cold	1. Inhibition of the like TNF-α, IL- 1, IL-6, and IL-10. 2. Inhibit adhesion molecules (ICAM-1, P-selectin) and NF-κB expression	1. Prevention of Viral Pathogenesis 2. Alleviation of the symptoms

*Certain nutraceuticals have inhibitory effects against a range of viruses in animal and human studies. With the advent of a novel coronavirus strain, nutraceuticals pose as a safer and an efficient substitute to help in offering the relief in those infected with encapsulated RNA viruses*.

### Resveratrol

Resveratrol or 3,4′,5-trihydroxy-*trans*-stilbene is a recognized phytoalexin class of nutraceuticals (generally produced in the presence of stimuli like stress or pathogenic attack) and is a polyphenolic stilbene compound mainly found in the fermented products derived from spermatophyte family of plants such as grapes (red wine), mulberries, and peanuts. In addition to being a useful compound in the treatment of cardiovascular diseases and cancers and as a promising agent for enhancing longevity (by scavenging superoxide, hydroxyl, and lipid hydroperoxyl radicals), it has a broad spectrum of antiviral effect with proven potential *in vitro* and *in vivo*. It acts by attenuating the generation of superoxides in the mitochondria and stops arachidonic acid-induced mitochondrial dysfunction. It also inhibits virus protein production, gene expression, and nucleic acid synthesis at multiple levels (5). The antiviral properties of resveratrol have showed positive results when tested on several viruses such as influenza virus, hepatitis C virus (HCV), respiratory syncytial virus (RSV), varicella zoster virus, Epstein–Barr virus, herpes simplex virus (HSV), HIV, and African swine fever virus, and their details have been summarized in Table 1. However, in the case of HCV and multiple sclerosis (MS), the progression of disease worsened after RSV administration. Dose-dependent addition of resveratrol in the HCV replicon system OR6 *in vitro* significantly enhanced HCV RNA replication. Similarly, the *in vivo* study of resveratrol in mice on a viral model of MS, named autoimmune encephalomyelitis (EAE) worsened the condition of mice as compared to the control group (10, 11, 15).

### Quercetin

Quercetin chemically belongs to the bioflavonoid group of nutraceuticals (flavonol), which can be widely found in fruits, vegetables, and tea. It has a broad range of actions such as signal pathway modulation, antimalignancy, antiviral, anti-inflammatory, and antioxidant. The antiviral property of quercetin possesses a wide spectrum in nature as it can be effective against both DNA (e.g., herpesvirus) and RNA (e.g., coronavirus and influenza) viruses. Quercetin can inhibit the viral DNA replication and can also affect the postviral healing by interacting with signaling pathways associated with post-transcriptional modulators. A few studies showed that quercetin in combination with resveratrol suppressed the secretion of pro-inflammatory mediators and chemokines in duck enteritis virus and human metapneumonia virus-led infections, thus minimizing cell oxidative damage (6). A study demonstrated the effectiveness of quercetin in hindering the replication cycle of parainfluenza virus-type 3 (Pf3) by inhibiting its DNA replication *in vitro* (12).

### Curcumin

Curcumin (diferuloylmethane) is a polyphenolic group of nutraceuticals, which can be easily obtained from the rhizome of turmeric (*Curcuma longa*) in an abundant amount. Turmeric has been already used as a traditional medicine in Indian and Chinese civilization. In recent times, curcumin becomes a compound of interest to the scientists due to its potential medicinal effects. It is a highly pleiotropic molecule and has well-known antitumor, antioxidant, hypoglycemic, wound healing, anti-inflammatory, antiviral, and anti-infectious properties and further research is still going on (7, 16). The molecular docking method demonstrated that curcumin binds to the target receptors, which are involved in virus infection mechanisms like spike glycoprotein-RBD, PD-ACE2, and SARS-CoV-2 protease, thus blocking virus entry and budding. The study also revealed that curcumin could possibly block ACE2 (a cell receptor, which binds to SARS-CoV-2 spike glycoprotein) to suppress novel coronavirus entry to the cell. Direct incubation with curcumin is said to reduce the ability of enveloped viruses to infect the cells as the former binds to viral surface glycoproteins and inhibits their activity (17). In another study, curcumin administration (50 and 150 mg/kg) *via* oral gavage in an animal model *in vivo* reduced influenza A virus (IAV) replication and lung injury (18). So far, ~300 clinical trials have indicated toward the usefulness of curcumin against cardiovascular, neurological, cancer, liver, metabolic, pulmonary, and inflammatory diseases. Coronavirus-induced “cytokine storm” results in a multi-organ failure. Curcumin blocks the necessary regulatory signals that are involved in several pro-inflammatory cytokines expression such as MAPK and nuclear factor-κB (NF-κB) pathways. Curcumin prevents inflammation and lung fibrosis by lowering the expression of vital cytokines and chemokines (IFNγ, MCP-1, IL-6, and IL-10) which are involved in viral infection (19). Despite several health promoting benefits, unstability and low bioavailability *in vivo* are the main factors, which limit the use of curcumin for clinical use on a wider scale. However, the use of other curcuminoids (demethoxycurcumin and bisdemethoxycurcumin), curcumin derivatives and synthetic curcumin analogs, liposome-encapsulated curcumin, curcumin-loaded apotransferrin nanoparticles, and nanoemulsions have increased cellular uptake, solubility, stability, and biological activity (18).

### Epigallocatechin Gallate

Flavonoids have been proven to be a useful functional medicine against a number of diseases. EGCG is a common catechin flavonoid, which can easily be found in tea and tea products. EGCG and its esters have encompassed several activities like anti-inflammatory, antibacterial, antiviral, antidiabetic, antihypertensive, etc. EGCG has reported to be helpful against a variety of viruses such as HSV, HCV, enterovirus (EV), and HIV. Besides this, a study demonstrated the capability of EGCG to act against influenza virus by blocking the nucleo-cytoplasmic movement of viral ribonucleoproteins in Madin–Darby canine kidney (MDCK) cells in a dose-dependent approach. (8) It has been hypothesized that EGCG, a zinc ionophore with substantially lower toxicity, can provide a positive effect similar to chloroquine (CQ) by increasing intracellular Zn^2+^ concentration, thus mediating its antiviral effect against SARS-CoV-2. However, human clinical trials need to be done to support *in vitro* studies and to establish the efficacy of EGCG (20).

### N-Acetyl Cysteine

N-acetyl cysteine is a prodrug, which is primarily employed as a mucolytic medium and also in the control of acetaminophen poisoning. The antioxidant and anti-inflammatory effects of this compound play an essential role in antiviral activity. Though the mechanism of action of the antiviral activity of NAC is still not fully discovered at a molecular level. However, it has been established that it is not only active against viruses like HIV and other viruses having a similar replication mechanism (which are dependent on nuclear transcription factors for their infectivity) but also against other viruses having a complete different pathogenesis of human diseases. It is a well-known fact that the lack of oxygen in a host cell environment, increased cellular stress due to the absence or less amount of GSH and more free radicals, secretion of inflammatory signal molecules play an important part in the virus pathogenesis, and like a GSH-replenishing prodrug, it helps body to fight against those viruses (9).

### Palmitoylethanolamide

Palmitoylethanolamide can be procured from the plant as well as animal sources. It is a cannabinoid receptor-inactive endocannabinoids (eCB)-related molecule, which is mainly used in prophylaxis for helping in the prevention of respiratory viral infection. It is recognized for its regulatory activity in cellular and metabolic homeostasis, antioxidant, anti-inflammatory, and immune-modulating capabilities (15). Its anti-inflammatory and antioxidant properties help in promoting an antiviral effect on the different types of viruses, especially common cold and influenza. It has also been used in Spain and Italy under the brand name Normast and in the earlier Czechoslovakia under the brand name Impulsin till 2008 while it is currently known as PeaPure. In the USA, it is sold as Recoclix for inflammatory bowel syndrome. It is also effective against various autoimmune disorders, like inflammatory diseases of the CNS and inflammatory bowel disease (10). An early randomized controlled trial (RCT) conducted on 468 healthy adults demonstrated that PEA administration lowered (45.5%) the incidences of headache, fever, and sore throat in comparison to the placebo group (*p* < 0.05). In a prophylactic trial with 918 participants, sickness days were reduced by a decrease of 40 and 32% after 6 and 8 weeks, respectively, relative to placebo (*p* < 0.0005). In another trial (901 volunteers) of postoral administration of PEA, a notable decrease in acute respiratory diseases (22.7%) and influenza virus titers was observed to be 34.4% in the placebo group (*p* < 0.0002) (14).

## Potential Nutrients for Prevention of Viral Infections

There are several natural compounds available, which have shown an antiviral potential in averting or/and debilitating viral diseases or for therapeutic applications. A nutrient-rich diet can lessen the possibility of chronic diseases and helps in making many viral infections less severe. Nutrients collectively comprise highly potent vitamins (vitamins A, D, C, E, B_6_, and B_12_), minerals (calcium and magnesium), trace elements (zinc, copper, selenium, etc.), carbohydrates, proteins, fats, and water. These multi-nutrients provide the highest nutritional value for all systems of the body, including bone, cardiovascular, liver, skin, and immune support. Therefore, eating a balanced diet rich in multi-nutrients can improve immunity in addition to maintaining respiratory and pulmonary health.

### Role of Vitamins in Antiviral Immunity

Vitamins brace the immune system of the body at three different levels, i.e., physical barrier (such as mucous membrane and skin), antibody production, and cellular immunity. Vitamins C and E help in strengthening the physical barriers. Vitamins C, D, and E assist immune functions at a cellular level. In addition, vitamin C is involved in antibody production. Table 2, adopted from Wikefeldt (21), provides a general summary of the common sources of nutrients along with their role in body functions, which have been further discussed in the following sections.

**Table 2:** Functions of immunity building nutrients in the human body along with their food sources.

**Nutrient**	**Function**	**Food Sources**
Vitamin A	Helps in maintaining mucosal lining of the gastro-intestinal and respiratory tract, regulates innate immunity, production, growth and differentiation of antibodies and lymph cells, anti-inflammation vitamin, inhibit apoptosis.	Orange and yellow fruits, Citrus fruits, sweet potatoes, carrots, bell pepper, dark green leafy vegetables etc.
Vitamin B6	Helpful in fighting against infection by supporting various biochemical reactions in the body, involved in nerve function and antibodies production, communicative interactions between cytokines and chemokines	Whole grains, beans, avocados, sunflower or sesame seeds, pistachios, fish, carrots, fish, milk, rice, and onions
Vitamin C	Antioxidant, protects cells from damage, besides boosting bone and tissue growth, helps in proper working of the immune system by regulating activity of T-lymphocytes and phagocytes, regulates drug metabolization.	Citrus fruits, papaya, spinach, kale, Brussel sprouts, broccoli, tomato, cabbage, cantaloupes, green peas, green, and red pepper.
Vitamin D	Needed for healthy bones, muscles and nerves fibers, regulates innate adaptive immune system response to identify and destroy pathogens.	Fatty fish, egg yolk, liver, mushrooms, fortified milk, juice or cereal, and sunlight (for synthesis)
Vitamin E	Antioxidant, protector of proteins and membrane fatty acids, modulate host immune functions, regulates humoral, and cell-mediated immunity.	Sunflower and safflower seeds, avocados, squash, almonds, peanuts, spinach, tomato, kiwifruit, trout, shrimp, olive oil, wheat germ oil, and broccoli
Protein	Building blocks and required in the production of antibodies and complement proteins	Low-fat dairy products, milk, yogurt, and cottage cheese, beans, brown rice, soy products, nuts, beans, chia seeds, chickpeas, peanuts

*As per WHO, nutrients are vital for disease prevention, management of health conditions, growth, and good health. Majority of nutrient quota for the body is met from the food, which comprises fruits and vegetables (21)*.

#### Vitamin A (Retinol)

Vitamin A is also recognized as an anti-inflammatory vitamin due to its crucial role in promoting the response of the immune system. It plays a regulatory role in the humoral and cell-mediated immunity through surface IgA, T helper cell modulation, and the generation of cytokines. Vitamin A has shown a proven therapeutic significance in the therapy of several infectious diseases such as measles-associated pneumonia. The supplementation of vitamin A has been widely researched as a part of the potential supporting the therapy for the prevention against the occurrence of acute lower respiratory tract infections (ALRTIs) and reducing the severity and for a speedy recovery. It has been found that children suffering from the deficiency of vitamin A tend to be at a larger risk of death and illness because of respiratory tract infections (15). Furthermore, a meta-analysis study showed that the infection worsened in children with preexisting vitamin A deficiency and its supplementation has been displayed to lower the risk of death by ~23–30% in 6–59-month old children. Vitamin A can be taken from orange/yellow fruits and vegetables. Permissible dose, as suggested by researchers, is up to 10,000–25,000 IU/day (22).

#### Vitamin D (Ergocalciferol)

Vitamin D is a steroid hormone and an immune system modulator, which lowers the expression of inflammatory cytokines in addition to increasing the macrophage activity. It also promotes the expression of antimicrobial peptides (AMPs) that are present in natural killer cells, monocytes, neutrophils, and epithelial cells lining the respiratory tract (23). Vitamin D acts both by suppressing and defending against infection by increasing anti-pathogen peptides. A few studies suggest the role of vitamin D supplementation in preventing infections in the upper respiratory tract. It also modulates transforming growth factor-beta (TGF-β) and reduces cytokine expression, thus favorably modulating virus-induced pathological cellular processes (24).

However, the research is limited to laboratory scale, is not established clinically, and shows that vitamin D (increased IL-1β in cell culture) plays an essential role in fighting viral infections. The study suggested that a range of >50 and <80 ng/ml serum 25-hydroxy vitamin D might prove helpful in mitigating morbidity from COVID-19. Current reviews recommend that vitamin D modulates innate responses of the immune system to respiratory viral infections, like RSV, parainfluenza 1 and 2, and influenza A and B (15). Martineau et al. (24) performed an RCT on 11,321 different people of 14 countries and noticed that vitamin D intake significantly lowered the instance of respiratory infections in people already having deficiency besides lowering infection risk in those with sufficient levels of vitamin D. Another study demonstrated the use of vitamin D in improving the response to antiviral treatments in patients suffering from HIV and hepatitis C (25). A study conducted by the Journal of the American Geriatrics Society (2016) showed that elderly patients who were given significantly higher doses of the Vitamin D3 had 40% fewer chances to attain lung infections. Deaths in older people are higher due to infections like bronchitis, pneumonia, and influenza because of their weakened immune function. According to research, vitamin D helps in strengthening the first line of defense with age, thus preventing chronic respiratory infections. About 1,000–4,000 IU of vitamin D/day intake is enough though people with severe deficiency need much higher doses (26).

#### Vitamin C (Ascorbic Acid)

Vitamin C assists several activities of both innate and adaptive immune system at a cellular level. It gathers in the phagocytic cells of the immune system, like neutrophils, and promotes microbe killing through chemotaxis, phagocytosis, the production of reactive oxygen species (ROS), and finally microbial killing. Vitamin C supplements hold the potential to prevent and cure systemic and respiratory infections by strengthening immune functions and therefore are actively used in hospitals to treat SARS-nCoV-2 infection too. Permissible dose, as suggested by researchers, is up to 1–3 g (one tab by mouth once a day) (27). Prophylactic approaches to prevent infection emphasizes daily dietary intake of vitamin C, which provides enough if not saturating plasma levels (100–200 mg/day). On the other hand, for the treatment of developed infections, a much higher dose (g) is required to recoup for a higher metabolic requirement (28). A scientific work in 11,306 people comprising 29 studies showed that daily vitamin C supplementation at a dosage level of 1–2 g/day decreased the interval of cold by 14% in children and 8% in adults (29). Furthermore, intravenous vitamin C when administered with high doses improved symptoms in people suffering from serious infections, viral infections-induced sepsis, and acute respiratory distress syndrome (ARDS) (30). However, at higher doses, vitamin C daily can lower the level of copper in the body, especially in people with a copper deficiency, which can in turn adversely affect immune function. A recent study posted to clinical trials by Peng (31) of Zhongnan Hospital, China stated the conduction of vitamin C infusion treatment for the therapy of serious 2019-nCoV infected pneumonia on 140 patients. Another randomized controlled experiment was recorded lately in the Chinese Clinical Trial Registry and conducted on Vitamin C and COVID-19 signifying the importance of vitamin C tablets in combination with diammonium glycyrrhizinate enteric-coated capsules in the treatment of novel coronavirus-caused pneumonia (32). Coronavirus (2019-nCoV) infection induces a cytokine upsurge, which leads to excessive inflammation and consecutively collateral lung damage and higher mortality. Conclusively, vitamin C infusion, an antioxidant, may be used as a symptomatic supportive treatment to help fight against oxidative stress and inflammation. A meta-analysis represented that the length of mechanical ventilation was shortened by 14% in the group that received vitamin C infusion. Several trials on humans, animals, and cells have confirmed the antiviral potential of vitamin C. Furthermore, vitamin C has shown promising results in controlled trials, by lowering blood pressure, decreasing bronchoconstriction, improving endothelial function, lowering the incidence of atrial fibrillation, evading pain, shortening the span of colds, and their incidence in physically worn out adults in addition to having potential beneficial effects against pneumonia (29).

#### Vitamin E (α-Tocopherol)

Vitamin E is an antioxidant with the potential to regulate the host immune response, and its insufficiency is known to hamper humoral and cell-mediated immunity. A study in elderly patients showed that vitamin E supplementation (200 IU/day) did not have much impact on lower respiratory tract infection but offered a shielding effect on upper respiratory tract infections, in particular common cold (33). The positive effects of vitamin E supplementation positively affected the medication of chronic hepatitis B observed in a small pilot randomized control trial, where a significantly higher normalization of liver enzymes and hepatitis B virus-DNA negativization were noticed in the vitamin E group. Similiar results were noted in a RCT in pediatrics, wherein vitamin E treatment led to a higher anti-HBe seroconversion and virological response (34). Although one RCT depicted that neither everyday multivitamin-mineral supplement nor vitamin E (200 mg/day) intake depicted a beneficial result on the occurrence and severity of acute respiratory tract infections in well-nourished adults. On the other hand, vitamin E supplementation showed increased severity and symptoms, illness duration, and activity restriction in the group (35).

#### Vitamin B Complex

Vitamins falling under the B complex group play an important role in the proper functioning of the human body including an improvement in the respiratory function activation of the innate and adaptive immune responses and maintenance of endothelial integrity. The administration of high doses of vitamin B_1_ or thiamine in patients at early stages of COVID-19 facilitates antibody responses and has the potential of limiting hypoxia. A study on combination of vitamin B_2_ (riboflavin) and UV light not only reduced the level of SARS-CoV-2 in human blood but also acted against MERS-CoV virus (36). Vitamin B_3_ (niacin) acts as a precursor of NAD and NADP, which acts by reducing pro-inflammatory cytokines (IL-1β, IL-6, and TNF-α) and also possesses immunomodulatory properties. It reduces the inflammation in a patient suffering from a ventilator-induced lung injury. Moreover, niacin also prevents the replication of viruses such as vaccinia virus, HIV, hepatitis B virus, and EV. The deficiency of vitamin B_6_ or pyridoxine leads to immune dysregulation. A few studies show that low levels of pyridoxine have been observed in COVID-19 patients with high inflammation. Another study on vitamin B_9_ or folic acid showed that it was capable of inhibiting the enzyme, furin, thus preventing binding by the SARS-CoV-2 spike protein ultimately to hinder cell entry and virus turnover (37). Similarly, a recent work on vitamin B_12_ (cobalamin) suggested that an intake of methylcobalamin possesses the ability to lower the risk of COVID-19-associated organ failure. Intramuscular administration of the methylated form of cobalamin in patients suffering from its deficiency dramatically restored CD8+ lymphocyte production and increased CD4/CD8 ratio, CD3-CD16+, and CD16 CD57 count in turn boosted the NK cell activity. Further, the reduced level of vitamin B_12_ has also been reported in COVID-19 patients (38).

### Role of Trace Elements and Minerals in Antiviral Immunity

A general summary of conventional sources of minerals and trace elements along with their role in body functions is discussed in the following sections as listed in Table 3.

**Table 3:** Functions of immunity building trace elements and minerals in the human body along with their food sources.

**Name**	**Function**	**Food sources**	**References**
Zinc	Imperative in wound and scar healing, neurocognitive health, development of innate and adaptive immune response against invading viruses and bacteria.	Shellfish, toasted wheat germ, spinach, cashews, pumpkin, sesame seeds, squash, baked beans, chickpeas, dark chocolate	(39)
Copper	Required for maintaining nerve cells, lung elasticity, metabolism, making red and white blood cells, development and differentiation of immune cells	Oysters, organ meat, nuts, seeds, shitake mushrooms, lobster, liver, yeasts, black pepper, potatoes, leafy greens, and dark chocolate.	(40)
Selenium	Protects against oxidative damage and infection, thyroid gland functioning, key nutrient in counteracting virulence development, enhances vaccine responsiveness, development and differentiation of immune cells, delayed-type hypersensitivity activity	Milk and other dairy products, nuts, sea foods, organ meats, cereals, and grains	(41)
Magnesium	Regulates biochemical reactions, glucose levels, protein synthesis, neurological and muscle functions, regulates innate immunity, anti-inflammatory, antibody synthesis.	Almonds, spinach, roasted cashews, peanuts, soy milk, avocado, brown rice, yogurt, beans, banana.	(42)

*Like vitamins, minerals and trace elements are classified as nutrients, which support several bodily functions, including immunity optimization, and energy production. Antiviral properties of some of the minerals (magnesium) and trace elements (zinc, copper, and selenium) have been backed with strong scientific evidence*.

#### Zinc

Zinc acts by favorably modulating an innate and adaptive immune response and virus supported pathological cellular activities through attachment and multiplication. Zinc deficiency is common, and its supplementation is proven to prevent both the duration of viral infections and their severity. Zinc lessens the possibility of lower respiratory tract diseases, which might be of use as far as COVID-19 is concerned. Researchers suggest an intake of zinc (30–60 mg/day) in the form of citrate, glycinate, zinc acetate, orally and zinc gluconate as lozenges (43). Zinc supplements display mitigating effects against several common cold viruses and might also be helpful for patients who are already ill. One of the studies conducted in 64 hospitalized children suffering from ALRTI showed that the administration of 30 mg zinc daily lowered the infection duration by ~2 days as compared to the control group (44). Moreover, long-term zinc intake is considered generally safe for healthy adults (set upper limit, 40 mg). On the contrary, excessive prolonged doses may obstruct the absorption of copper, thus compromising the immune system. Many *in vitro* and clinical studies signify the effectiveness of zinc in eliciting antiviral activity. Shida (45) demonstrated that zinc had a strong effect on numerous respiratory viral infections by the modulation of the entry of viral particles, replication, fusion, viral protein translation, and its release. In another study, low Zn concentration increased the susceptibility to pneumonia, and subjects having a high serum Zn level (>70 μg/dl) were considered to be at a lower risk of getting pneumonia (*p* < 0.001), along with lower mortality and disease duration in comparison to the low-zinc group (<70 μg/ml). In addition, serum zinc concentration was reported to be 15% lower in the incidences of community acquired pneumonia (46). Zinc ions might have an anti-inflammatory activity in pneumonia, thereby minimizing oxidative stress and protecting the lungs against damage in sepsis and systemic inflammation. Zinc deficiency in the murine model of polymicrobial sepsis led to higher NF-κB p65 messenger RNA (mRNA) expression in lungs, which caused the upregulation of the target genes of TNFα, IL-1β, and ICAM-1. On the other hand, taking zinc supplements produced a protective effect in the lungs in the septic state with the help of the modulation of NF-κB and ERK 1/2 by lowering neutrophil infiltration and oxidative damage (47). The observed study also displayed that zinc stops the activity of RNA-dependent RNA polymerase (RdRp) of hepatitis E virus, ultimately affecting its replication. Zinc reportedly inhibits coronavirus RdRp activity as well *in vitro* and zinc ionophores to restrict coronavirus replication. Even though the antiviral properties of zinc have been well-established in addition to the possible property of CQ/hydroxychloroquine (HCQ) acting as a zinc ionophore, the synergistic effects of zinc with any one of these drugs still need to be confirmed. It is being speculated that high intracellular zinc concentration might also cause more proper RdRp obstruction resulting in more hinderance of intracellular SARS-CoV-2 multiplication (48).

#### Copper

Copper aids in the development and differentiation of the cells of the immune system. *In vitro* studies have also depicted the antiviral properties of copper. Copper can kill various viruses such as HIV-1, bronchitis virus, poliovirus in addition to other enveloped and non-enveloped, and double- and single-stranded DNA and RNA viruses. Virus killing by copper might be mediated through ROS (49). Intracellular copper has been proven to regulate the influenza virus life cycle while copper-thujaplicin complex inhibits the replication of human influenza viruses. Turnlund et al. (50) studied the impact of prolonged high copper consumption on the immune system function in young men. They concluded that benzylamine oxidase, superoxide dismutase, and plasma ceruloplasmin activity were more in amount when the copper intake was 7.8 mg/day, as compared to an intake of 1.6 mg/day, thus showing an enhancement in the antioxidant level (50). Though a significant reduction in the percentage of serum IL-2R, circulating neutrophils, and the antibody titer was observed at a higher copper intake (7.8 mg/day) against the Beijing strain of influenza (4). The literature also illustrated that the exposure of copper to nCoV 229E irreversibly affected the morphology of virus by breaking it into envelope and dispersing surface spikes in addition to destroying the viral genome (51). A cell-based study proved that copper ions were able to block the protease-2 required by SARS-CoV-1 for its replication. Another study conducted in China on 71 adults suffering from COVID-19 found that all participants had a relatively low serum total cholesterol level in comparison to healthy adults. However, several studies have also indicated the link between a low level of total cholesterol to the reduced concentration of copper (52). Thus, copper supplements might also prove helpful in the fight against novel viral diseases like COVID-19 through extensive clinical data, and experimental results may be required to support the same.

#### Selenium

Selenium is an important mineral for a healthy immune system. Research on animal models indicates the potential of selenium supplementation in increasing the antiviral response against various influenza strains, such as H1N1. A few studies on the virulent strains of influenza and coxsackie viruses demonstrate that the acute deficiency of selenium can enhance disease severity and pathogenicity by promoting numerous mutations in the viral RNA. Therefore, selenium is essential both for boosting host antiviral (Th1-type) immunity and for obstructing the evolution of some viral pathogens into more virulent strains. Reduced concentration of selenium and selenoenzymes [such as thioredoxin reductase (TrxR) and glutathione peroxidase (GPx)] in erythrocytes and plasma has been observed in children afflicted with highly virulent H1N1 subtype of IAV (53). In a French RCT conducted on adults having low plasma selenium levels were given 20 mg Zn and 100 mg Se supplements for 15–17 months. It was found that adults receiving supplementation displayed a better humoral defense post-influenza A vaccination in comparison to the adults from the placebo group (54). IAV-infected tissues and cells may be safe guarded from virus-led oxidative stress and cell death with the optimized activity of GPx under the conditions with adequate selenium. Also, according to a study, bronchial epithelial cells cultured in a Se-deficient environment showed more cell death due to apoptosis after being infected by IAV in comparison to cells grown in Se-adequate medium (55).

#### Magnesium

Magnesium is vital in regulating the immune function by exerting an influence on antibody synthesis, antibody-dependent cytolysis, immune cell adherence, immunoglobulin M lymphocyte binding, T helper-B cell attachment, and the response of macrophage to lymphokines. It acts as both an anti-inflammatory and a bronchodilator and has been used in a successive manner to clear the airways and make it easier to breathe. Some *in vivo* and *in vitro* studies emphasize upon the importance of magnesium supplementation in developing the immune reaction against viral infections (56). One of the studies showed that reduced concentration of free intracellular Mg^2+^ is responsible for an impaired expression of receptors of CD8+ T cells and natural killer cells (NKG2D) which in turn affects the cytolytic action against viruses and immune surveillance (6). Two patients suffering from EBV were given oral supplements of magnesium for 175 days in the form of magnesium threonate and magnesium sulfate, on patients suffering from severe EBV infection. Within 2 days of administration, an increase in NKG2D expression and free [Mg^2+^] ions was observed in patients along with a decrease in the number of EBV-infected cells. This implies that [Mg^2+^] ion homeostasis in the body is essential for NKG2D expressed CTLs, NK cells, and γδ T cells, which in turn mediate antiviral and antitumor immunity (56).

## Multi-Nutrient Supplements for Viral Infections

The deficiency of micronutrients in the body weakens the immune system by affecting the adaptive antibody response and T-cell-mediated immune response, subsequently causing a balanced host response dysregulation. Certain trace elements and vitamins support immunity by strengthening epithelial lining as well as cellular and humoral immune responses. Moreover, trace elements and vitamin supplement in various combinations have shown favorable results on the antiviral ability of immune response. In a randomized clinical trial comprising 725 elderly patients, considering the parameters such as humoral response to influenza vaccine, delayed-type hypersensitivity skin response, infectious mortality, and morbidity depicted that adequate zinc supplementation in combination with selenium increased humoral activity post immunization relative to the control group. A study on older adults showed that multi-nutrient supplement comprising a combination of trace elements, such as selenium sulfide and zinc, vitamins like ascorbic acid and β-carotene, corrected specific nutrient deficiencies within 6 months of administration. Respiratory tract infections were absent in patients who received trace elements. In addition, it was observed that the level of antibodies after influenza vaccine administration was higher in groups, which received trace elements alone or were associated with vitamins as compared to the group that received vitamin alone with lower antibody titers (54). One study conducted on 878 patients suffering from HIV subtype C having a higher cell count than the normal (>350/μl) and underwent antiretroviral therapy revealed that the supplementation of vitamin E plus selenium, vitamin B complex, and vitamin C helped in slowing down the disease progression in addition to lowering the morbidity (57).

## Role of Milk Proteins and Peptides in Antiviral Immunity

Protein imbalance in the diet leads to malnutrition resulting in impaired immunity, particularly affecting the T-cell system, leading to increased opportunistic infections and mortality in hospitalized patients. Active milk proteins and peptides possess antiviral and immune regulating characteristics (58). Human and bovine lactoferrin (bLF), lactoperoxidase, artificially altered milk proteins such as serum albumin, β-lactoglobulin, and α-lactalbumin act on viruses like HIV, by binding to their cellular receptors, thus inhibiting viral absorption and ultimately replication. Lactoferrin can effectively bind to the heparan sulfate and mannose receptor of HIV, which inhibits virus attachment (59). Another work demonstrated the usefulness of lactoferrin in strongly inhibiting viral reverse transcriptase. Meanwhile, α-lactalbumin, β-lactoglobulin, and casein were effective in strongly inhibiting the protease and integrase of HIV (60). Lactoferrin of both human and bovine origin has been depicted to show an antiviral response against a wide spectrum of viruses such as influenza virus (H1N1, H3N2, and H5N1) dosage, esterified bLF <20 mg/ml. The antiviral response of lactoferrin against parainfluenza virus and RSV is also demonstrated by inhibiting viral replication (61). Even the methylated forms of β-lactoglobulin, α-lactalbumin, and lactoferrin elicit the antiviral response against human influenza virus A, H3N2, H1N1, and lethal avian IAV (H5N1). The antiviral response is linked to the binding of whey proteins to virus hemagglutinin, viral DNA and RNA, and is disrupted, thereby making viral proteins unstable and incapable of their attachment to the cell membrane (62). One of the studies indicated that the intake of lactoferrin along with milk immunoglobulin reduces the occurrence of common cold in adults. Lactoferrin intake increases the NK cell response in adenomatous colorectal polyps patients. Therefore, lactoferrin might partially mediate protection to the host against influenza and common cold by increasing the number and activity of NK cells (63). Lactoferrin has also been classified as a host defense protein due to its ability to enhance cytotoxicity by increasing the functions of lymphokine-activated killer cells and NK cells particularly in infants. In addition to this, it is also involved in macrophage activation along with the stimulation of pro- and anti-inflammatory cytokine release IL-1, IL-6, IL-8, IL-18, IL-γ, and TNF-α. Further, a few studies on milk proteins and peptides have demonstrated synergy with drugs such as acyclovir, ribavirin, and zidovudine, against HSV, human HCV, and HIV 1, respectively, by reducing drug dosage, preventing the development of drug-resistant viruses, and selective targeting (64).

## Functional Foods for Enhancing Immunity

Functional food is not a single component; it is a combination of different nutrients that are high in a particular component imparting therapeutic benefits. Functional foods contain supplements or additional nutrient-rich ingredients, such as oats, a rich source of dietary fiber having a beta-glucan, or fiber-enriched vermicelli, which increase the immunity by reducing the inflammation, and thereby helps in improving the health (65). These constitute conventional (grains, fruits, vegetables, fermented foods, herbs, spices, beverages, and nuts) or wholesome natural foods as well as modified foods (yogurt, cereals, and orange juice) that can be fortified with vitamins, minerals, and probiotics for additional health benefits. In July 2002, about 300 food products were recognized as foods for specified health use (FOSHU) status in Japan. Similarly, a large quantity of antioxidants lying in vegetables and fruits also help in combating diseases (66). A few previous literature studies has also shown that fried food has impaired the white blood function and severely altered gut microbiota. Plant-based diets have been proven to be effective in reducing the risks for influenza and pneumonia as these are rich in dietary fiber, antioxidants, and vitamins (67), which lower body mass index (BMI), and thereby help in improving immunity. However, health benefits and claims associated with the consumption of these functional foods still need to be worked upon to establish a strong scientific proof regarding its safety and efficacy. Honey is one such functional food having a well-studied antiviral potential. Apart from sugars, honey contains several other minor components such as minerals, vitamins (majorly vitamin C), carotenoids, proteins, amino acids, enzymes (catalase and glucose oxidase), volatile compounds, and organic acids. The constituents impart several benefits to honey, which is possibly used in the treatment of diseases by anti-inflammatory, immunomodulating, phytochemical, antioxidant, antibacterial, antiviral, antitumor, and vasodilative activities. Flavonoid and phenolic compounds of proven therapeutic significance, such as gallic acid, ellagic acid, cinnamic acid, benzoic acid, caffeic acid, coumaric acid, apigenin, myricetin, quercetin, catechin, naringenin, and luteolin, are the main bioactive compounds present in honey, which also exert an antioxidant potential. Vitamin C and phenolic content together impart an anti-inflammatory effect. Evidence suggests that honey lowers the inflammatory action in cell cultures, animal models, and clinical trials. A tissue culture study indicated that honey enhanced the level of antibodies, T and B lymphocytes, neutrophils, eosinophils, monocytes and the generation of NK cell production during immune response (68). Manuka honey from New Zealand is said to possess antiviral activities against influenza virus (H1N1) strain A/WSN/33 in MDCK cells. Another *in vitro* study also showed the effectiveness of commercial manuka honey against a HSV-1 isolate using Vero cells (69). Charyasriwong et al. (70) reported that an active ingredient, methylglyoxal, present in manuka honey showed an activity against H3N2, H1N1, H5N2, and also oseltamivir-resistant H1N1. Similarly, berries are rich in bioactive compounds, particularly polyphenolics, flavonoids along with polysaccharides, carotenoids, organic acids, anthocyanins, etc. have been used as a natural cure against upper respiratory tract infections. Elderberry extract exerts a regulatory influence on viruses both by directly obstructing viral glycoproteins and indirectly by increasing the expression of IL-6, IL-8, and TNF (71, 72). A concoction of raspberry extract, elderberry juice, and honey, named, Sambucol stopped the hemagglutination and multiplication of 10 different influenza type A and type B virus strains *in vitro*, which were isolated from both humans and animals. The therapeutic effect of Sambucol containing berry extracts may be due to the stimulation of pro-inflammatory cytokines (IL-6, IL-8, IL-1β, and TNF-α) production, and anti-inflammatory cytokine (IL-10) by macrophages as well (73). The administration of goji berries increased the activity of flu vaccine in adult mice in addition to increasing cytokine secretion and IgG titers, thereby modulating the immune response (TNF-α and IL-12). Increased maturity and expression of dendritic cells (CD40, CD80, and CD86) were also observed (74). Another research work reported that a black currant (*Ribes nigrum*) extract obstructed influenza type A and type B virus adsorption as well as the pandemic strain oseltamivir-resistant A/Yamagata/5/2009 and A/Yamagata/165/2009 pdm. Furthermore, a black currant extract also checked the factors involved in influenza infection such as pneumonia causing bacteria (*Streptococcus pneumonia*), *Haemophilus influenza*, and viruses like adenovirus (AdV) and RSV, which infect the respiratory tract (75). Krawitz et al. (76) observed that the administration of Rubini, a commercial elderberry extract preparation, inhibited the activity of pathogens responsible for respiratory diseases including influenza viruses (type A and type B). Similarly, cranberry juice and its polyphenolic component have been also revealed to inhibit influenza A and B strains lowering infectivity titers of rotaviruses and food-borne viruses like feline calicivirus and murinenorovirus (77). Polyphenolic fractions rich in polyphenolics extracted from bilberry (*Vaccinium myrtillus* L.), Natsuhaze (*Vaccinium oldhamii* L.), cranberry (*Vaccinium oxycoccos* L.), and procyanidin, fraction from Canadian blueberry (*Vaccinium angustifolium* L.) have also exhibited a significant antiviral activity against influenza viruses (78). A study disclosed that an aqueous extract of Korean black raspberry (*Rubus coreanus*) inhibited hepatitis B activity. A similar study on another raspberry species (*Rubus imperialis* L.) depicted anti-HSV1 activity *in vitro* (79). Therefore, berries rich in several bioactive components could be a vital raw material for new drugs. Though, more lab studies and clinical trials need to be undertaken to establish their antiviral efficacy.

## Role of Probiotics Supplements in Antiviral Immunity

Probiotics are live microorganisms that impart health benefits when provided in a sufficient quantity to the host. Bacteria from the genus *Lactobacilli* and *Bifidobacterium* are the most common probiotic microorganisms. Probiotics can be incorporated in the diet in the form of buttermilk, yogurt, bread, sourdough, tempeh, bread, kombucha, cottage cheese, fermented sauerkraut, fermented pickle, miso soup, and kimchi (80). A research work demonstrated that *Lactobacillus rhamnosus* GG (L. GG, ATCC 53103) is completely safe for consumption even by premature infants, indicating the potential of probiotics as a relatively safer option for therapy-based intervention in any age group (81). However, people possessing a weakened immune system due to chemotherapy and critical illness need to be cautious while using probiotic supplements. Specific probiotics have known to be useful in reducing the severity and duration of acute rotavirus-caused gastroenteritis and virus-originated respiratory tract infections.

Even though vaccines are promising prophylactics that are useful against viruses, but their efficiency is limited due to rapidly mutating viral RNA such as in the case of influenza virus. In this regard, probiotics needs to be widely researched to establish their efficacy as a part of antiviral supportive therapy as they have proven a virucidal action against many respiratory viruses as well (Table 4). The mechanisms by which probiotics act against respiratory viruses most likely are as follows: (a) by adhering to the epithelial layer, hence block the adherence of viruses by a steric hindrance; or by competing with them for specific carbohydrate receptors, (b) by directly inhibiting the attachment of virus to host receptor cells by binding to it, (c) likely by inducing mucosal regeneration; intestinal mucins may inhibit viral replication by inhibiting their adherence to epithelial cells, (d) by directly producing antimicrobial substances against pathogens, (e) by activating and modulating the immune response *via* dendritic cells and macrophages, (f) by inducing dehydrogenase and mild NO, lactic acid and dehydrogenase generation may have antiviral activities, (g) stimulating the immune system by IL, NK cells, Th1 activity, and IgA production (3, 89).

**Table 4:** Effect of different probiotic strains on viruses.

**Probiotic strain**	**Target disease/virus**	**Mechanism of action**
*lactobacillus casei* (yakult) (82)	Upper respiratory tract infection, Epstein–Barr virus (EBV), Cytomegalovirus (CMV)	Lowered plasma CMV and EBV immunoglobulin titers
*lactococcus lactis* jcm5805 (l. lactis plasma) (83)	Influenza	Reduction in the duration of cough and sore throat Increment in IFN-α mRNA in PBMCs
*lactobacillus rhamnosus* gg (84)	Rhinovirus infection	Slight reduction in the incidence and severity of cold symptoms
*l. plantarum* 06cc2 (3)	IFV A/PR/8/34 (H1N1)	Decrease in body weight, virus count in lungs and number of macrophages and neutrophils in bronchoalveolar lavage fluid (BALF), TNF-α in BALF, INF-α, IL-12, and IFN-γ Increase in activity of NK cell, IFN-γ in Peyer's patches and survival of mice
*l. plantarum* ncimb 8826 (85)	Pneumonia virus of mice (J3666)	Enhanced protection against virus infection Decrease in Granulocyte recruitment, CXCL10, CXCL1, CCL2, TNF, and virus recovery
*l. reuteri* f275 (86)	Pneumonia virus of mice (J3666)	Increase in neutrophil deployment, CXCL1, CCL2, CCL3, CXCL10, TNF-α, IFN-α, IFN-β, IFN-γ, and IL17A
*Enterococcus faecalis* fk-23 (87)	Hepatitis C virus	Significant reduction in alanine aminotransferase No significant difference in viral load
*Bifidobacterium animalis* (bb12) (88)	Intestinal Ig responses to rota and polio- virus in infants	Evident increment in fecal anti-poliovirus and anti-rotavirus specific IgA.

*Probiotics depict the ability to regulate and modulate the immune system. Some probiotic strains have shown an effective antiviral activity including respiratory viruses. However, the mechanism of action against viruses differs from one probiotic strain to another*.

### Probiotics for Upper Respiratory Tract Infections: Clinical Studies

The role of probiotics in preventing upper respiratory infections (URIs) has been extensively studied.

A specific interaction of probiotics with pathogens can potentially reduce their colonization in the nasopharynx, hence reducing URI and acute otitis media (AOM). The microbiota of an individual could be studied and integrated into healthcare to target his specific diseases for a better treatment. Yogurt consumption consisting of *Lactobacillus delbrueckii*s sp. bulgaricus OLL1073R-1 (R-1) triggered the activity of NK cell and probability of getting a common cold in the elderly was reduced (82, 89). Similar studies demonstrated the significance of secreted polysaccharides of R-1 in improving immune functions along with NK cell activation. Thus, R-1 or its products might play a role in preventing virus-induced respiratory infections (90). In another study, it was found that intranasal inoculation of *Lactiplantibacillus plantarum* or *Lactiplantibacillus reuteri* guarded against lethal infection caused by pneumonia virus in mice (85, 86). Furthermore, probiotics along with prebiotics have proven their efficacy in increasing immunogenicity by affecting seroprotection and seroconversion rates in the elderly administered with influenza vaccine (87, 91). Therefore, a vitamin-probiotic combination could act as a possible immunity booster in a generic manner. For example, vitamin D can modulate an adaptive and innate immune system as the vitamin D receptor is expressed on the surface of all immune cells and also all immunologic cells can produce a vitamin D metabolite (88, 92).

## Conclusion

For new viral diseases, no specific pharmacological treatment for their prevention or treatment can be made available immediately. In this regard, research focuses on strengthening the immune system by adopting nutritional strategies. This review aims to put forward the therapeutic and preventive potential of some nutrients, nutraceuticals, trace elements, milk proteins, peptides, functional foods, and probiotics. Further, an awareness about the use of nutrient fortified cereals and grains, nutraceutical products should be created as a part of disease preventive and health promotive approach. In view of the current pandemic of COVID-19, a type of severe acute respiratory infection and a deficit of effective targeted antiviral drugs exist thus a symptomatic support therapy is still the approach to be followed. An in-depth knowledge of the regulatory molecules involved in the molecular mechanism of epigenetic interaction and replication can promote the development of functional food products having an antiviral and immunity potential as a component of the effective therapeutic strategy. Therefore, it is crucial to study the clinical relevance and safety of compounds with proven immune enhancing properties for viral pneumonia as well through RCT. Likewise, zinc ionophores like quercetin and EGCG can act in a way similar to drugs such as CQ/HCQ by increasing intracellular Zn^2+^ levels without adverse effects. Clinical trials and *in vitro* studies can be done in this regard. Milk proteins and related peptides have enormous scope to be used as supplements, templates, and novel vaccine adjuvants for designing further potent antiviral drugs. Bioactive compounds such as nutraceuticals and functional foods with proven efficacy in hindering viral mechanisms, along with pharmaceutical medication in case of not being alone, might be instrumental in treating corona virus-induced infections. Although these supplements are beneficial for the immune system and health, they should not be used as an alternative to a healthy lifestyle, which is of utmost importance. Moreover, nutraceuticals are mostly food than medicine and act gradually, thus their long-term and regular ingestion is imperative in reaping the health benefits completely associated with them.

## Author Contributions

SS: investigation, visualization, formal analysis, and writing—original draft. PK: investigation, visualization, and writing—original draft. DK: resources, methodology, and visualization. VM: formal analysis, resources, and visualization. GS: resources, methodology, and formal analysis. KM: conceptualization, visualization, and writing—review and editing. PP: conceptualization, supervision, and writing—review and editing. MK: conceptualization, supervision, writing—review and editing, project administration, and funding acquisition. All authors contributed to the article and approved the submitted version.

## Conflict of Interest

The authors declare that the research was conducted in the absence of any commercial or financial relationships that could be construed as a potential conflict of interest.

## Publisher's Note

All claims expressed in this article are solely those of the authors and do not necessarily represent those of their affiliated organizations, or those of the publisher, the editors and the reviewers. Any product that may be evaluated in this article, or claim that may be made by its manufacturer, is not guaranteed or endorsed by the publisher.
